# Prediction of Hypoxic-Ischemic Encephalopathy Using Events in Fetal Heart Rate and Uterine Pressure

**DOI:** 10.22489/cinc.2023.380

**Published:** 2023-12-26

**Authors:** Johann Vargas-Calixto, Yvonne W. Wu, Michael Kuzniewicz, Marie-Coralie Cornet, Heather Forquer, Lawrence Gerstley, Emily Hamilton, Philip Warrick, Robert Kearney

**Affiliations:** 1 McGill University, Montreal, Canada; 2 University of California, San Francisco, USA; 3 Kaiser Permanente, Oakland, USA; 4 PeriGen Inc., Montreal, Canada

## Abstract

The objective of this work was to evaluate the utility of using intrapartum fetal heart rate (FHR) and uterine pressure (UP) events to detect infants at risk of hypoxic-ischemic encephalopathy (HIE). We analyzed data from 40,976 term births from three groups: 374 infants that developed HIE, 3,056 that developed fetal acidosis without HIE, and 37,546 healthy infants. We counted the transitions between FHR events and the length of FHR and UP events. Then, we used these features to train a random forest classifier to discriminate between the healthy and the pathological (acidosis or HIE) groups. Compared to the Caesarean delivery rates for each group, our system detected 6.9% more HIE cases (54.9% vs 61.8%, p<0.001) and 10.7% more acidosis cases (37.6% vs 48.3%, p<0.001), with no increase in the false positive rates in the healthy group (38.9% vs 38.8%, p=0.26). Importantly, over 3/4 of the HIE detections were made 3 hours or more before delivery. It is reasonable to expect that this would be enough lead time to permit clinical intervention to improve the outcome of birth.

## 1. Introduction

During labor, strong uterine contractions can limit the delivery of oxygen to the fetus, and lead to intermittent fetal hypoxia. The fetus is well equipped to deal with brief hypoxic periods through a series of anatomical and physiological adaptations. However, when the combination of degree, duration, and frequency of hypoxia overwhelms these mechanisms, it can cause brain injury and result in neonatal hypoxic ischemic encephalopathy (HIE). Moderate and severe forms of HIE can have catastrophic consequences such as cerebral palsy, motor or cognitive disorders, learning disabilities, and death [1]. In low- to middle-income countries, where HIE occurs in 6.7 to 26.5 cases per 1,000 births, 12 – 31% of infants with HIE die in the neonatal period [2].

It is standard of care in developed countries for clinicians to monitor fetal heart rate (FHR) and uterine pressure (UP) continuously to assess fetal tolerance to labor. Clinicians focus on recognizable events such as FHR baseline, accelerations, and decelerations, and the frequency of UP contractions. Unfortunately, the visual assessment of these events has high intra- and inter-observer variability [3], which hampers the ability to detect a fetus at risk of HIE. Moreover, the myriad of possible combinations of FHR patterns over time and their unclear relationship to hypoxia leads to considerable diagnostic uncertainty for clinicians and highly variable intervention rates. An automated method to assess risk of HIE could mitigate these problems. In this work, we used features of intrapartum FHR and UP events to train a classifier to detect fetuses at risk of acidosis and HIE.

## 2. Clinical data

Our database includes deidentified clinical, FHR, and UP data for 246,968 singleton births with a gestational age of at least 35 weeks and no congenital defects. This study was approved by the Research Ethics Boards of Kaiser Permanente and McGill University. The present study was limited to 40,976 births with umbilical cord or neonatal blood gas available within the first two hours of life, which provided an objective measure of the outcome of labor. These data were divided into three groups: (1) 37,546 healthy infants with blood pH > 7 and a base deficit < 10 mmol/L; (2) 3,056 infants that developed acidosis defined by blood pH ≤ 7, or base deficit ≥ 10 mmol/L, and no encephalopathy; and (3) 374 infants that developed HIE defined by the presence of acidosis and evidence of encephalopathy.

## 3. Methods

### 3.1. Preprocessing

Preprocessing of the FHR and UP was carried by PeriCALM Patterns for up to 12 h before birth. The PeriCALM Patterns software processes these signals to identify the events that clinicians assess at the bedside. Gaps shorter than 15 seconds were linearly interpolated. The FHR was sampled at 4 Hz and then filtered with low-pass, high-pass, and Karhunen-Loève filters. The UP was acquired at 1 Hz and upsampled to 4 Hz [4, 5].

### 3.2. FHR and UP events

PeriCALM Patterns identified the following FHR events using long short-term memory networks, and UP events using logistic regression [4, 5]:

1. FHR baseline (BAS): relatively flat segments of FHR typically in the 110 – 160 bpm range with a peak-to-peak variability of 5 – 15 bpm.
2. FHR accelerations (ACC): transitory increases in the FHR of more than 15 bpm lasting for more than 15 seconds before returning to baseline.
3. FHR decelerations (DEC): transitory FHR decreases of more than 15 bpm lasting for more than 15 seconds before returning to baseline.
4. Uterine contractions (CON): segments of increased UP before decreasing to a basal state.
5. Resting intervals (RIN): periods between contractions with little activity in the UP signal.

PeriCALM Patterns also identified uninterpretable segments, where the noise level and artifacts could prevent the FHR analysis. We divided the signals into 20-minute-long epochs, removing the noisy segments, and discarding epochs with less than 80% samples remaining. In each epoch, we extracted features of semi-Markov chains as previously defined in [6]. This involved counting the transitions between FHR events and the total dwell time of FHR and UP events, defined as the time spent in one event before transitioning into the next. There was a total of 11 features for each epoch: six FHR transition counts and five dwell times.

### 3.3. Classification Framework

We first divided the data for classification: 90% of individuals were used for training, and 10% for testing.

Using the training set, we trained a random forest (RF) classifier with 2,500 trees. The classification target was to discriminate between healthy and pathological epochs, which included both the acidosis and HIE group. RF samples with replacement from each class to train classification trees on different subsets of the data. We manipulated the sampling procedure such that the majority class was subsampled, and each tree was trained with a balanced number of observations from each class. The out-of-bag predictions of the training set were used for hyperparameter selection and validation. The RF predicted the probability $p_{HIE}$ that an epoch belonged to the pathological class. If $p_{HIE}$ was larger than a threshold $p_{t}$, an alert was issued for that epoch.

Finally, our system monitored the temporal progression of the alerts. If at any point in time, the last n consecutive epochs issued an alert, the system recommended intervention. A maximum of n/2 epochs was allowed. If more epochs were missing, the system did not decide until there were more observations. We examined values of n in the range 1–10. For each n we built a receiver operating characteristics (ROC) curve by varying $p_{t}$; we selected the value $\hat{n}$ giving the highest area under the ROC curve (AUC). Finally, we chose the value $\hat{p_{t}}$ that yielded a false positive rate (FPR) equal or lower than the Caesarean rate in the healthy class.

On the test set, we used the $\hat{n}$ and $\hat{p_{t}}$ to recommend interventions. We compared the rate of recommending intervention with the observed Caesarean rates of each group. We repeated the training and testing approach for 100 random samples of training and test sets to obtain distributions on the performance metrics.

## 4. Results

### 4.1. Epoch classification

**Figure 1:**
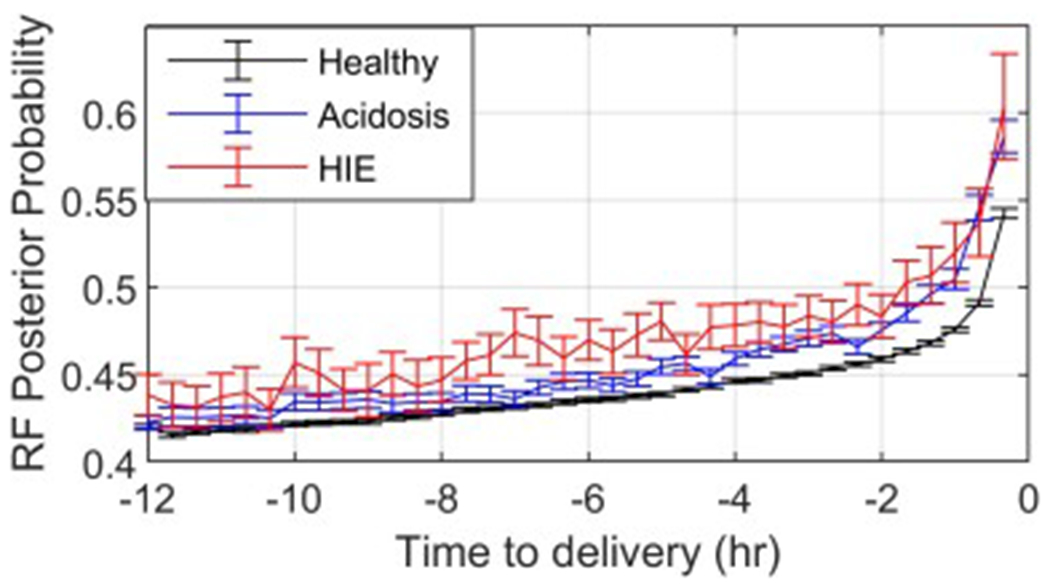
Median out-of-bag $p_{HIE}$ for the healthy (black), acidosis (blue), and HIE (red) groups as a function of the time before delivery. The bars display the 95% confidence interval of the median.

Figure 1 shows the median $p_{HIE}$, and its 95% confidence interval, as functions of the time before delivery for one iteration across all subjects in the training set. In all groups, $p_{HIE}$ increased as the time of delivery approached. The $p_{HIE}$ of the healthy group was always lower than that of the acidosis and HIE groups.

### 4.2. Cumulative predictions

**Figure 2:**
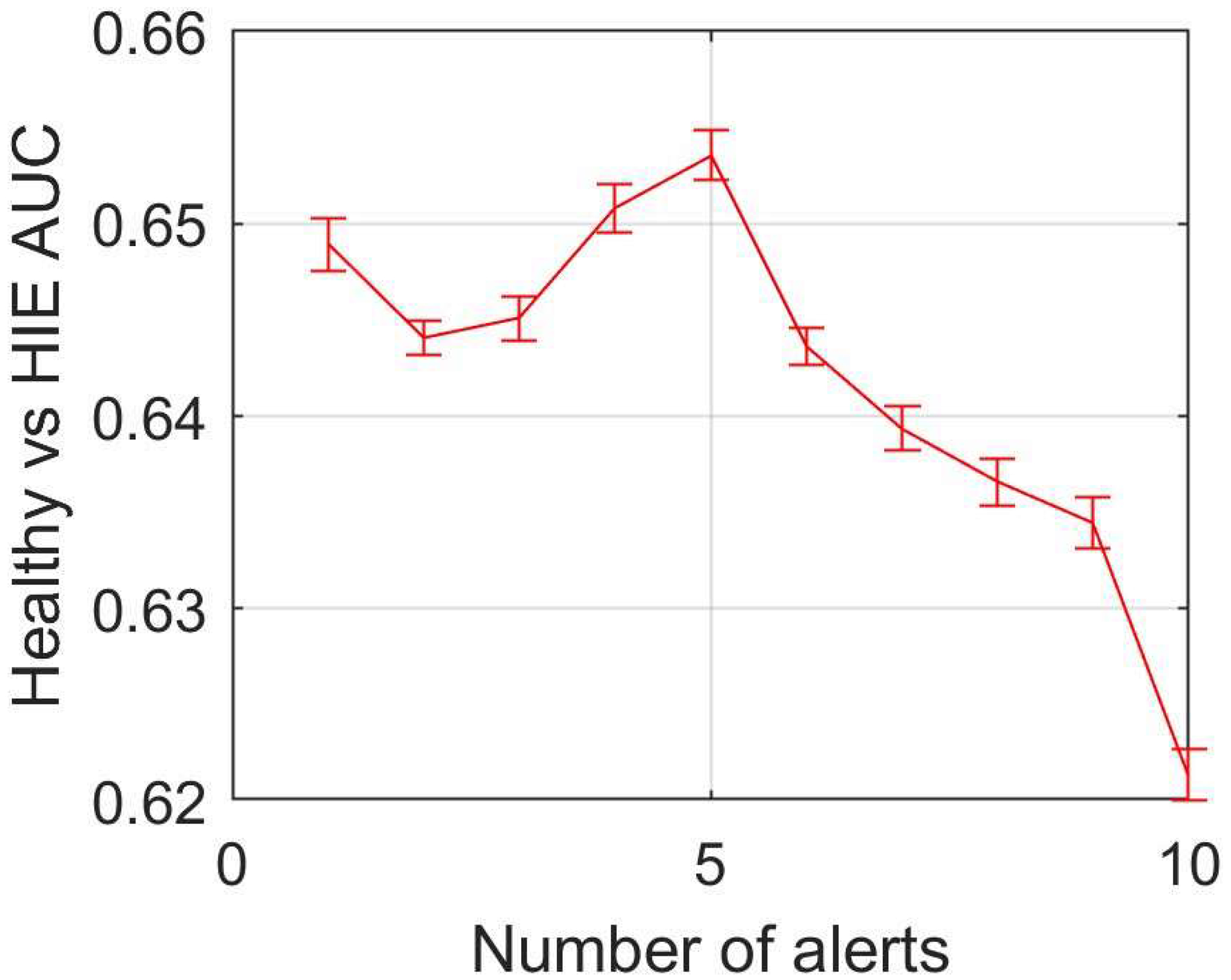
The median area under the ROC curve (AUC) as a function of the number of alerts. The bars indicate the 95% confidence interval of the median.

Figure 2 shows the median AUC, and its 95% confidence interval, of the recommendation system as a function of number of alerts. The AUC was maximal when $\hat{n}=5$ alerts were required.

**Figure 3:**
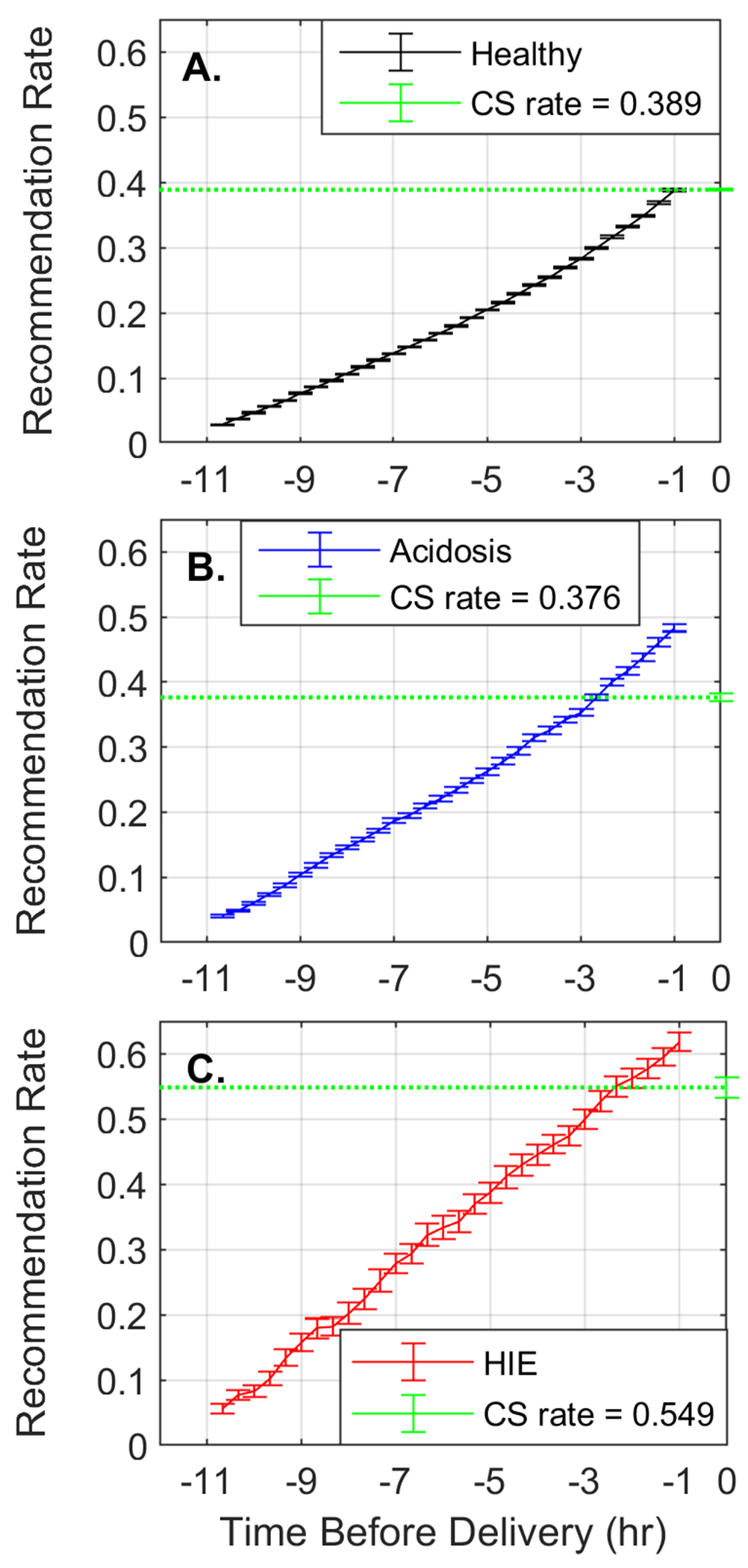
Our system recommendation rate in the (A) healthy (black), (B) the acidosis (blue), and (C) the HIE (red) groups, compared to the Caesarean delivery rates (green). The lines indicate the median performance and the error bars its 95% confidence interval.

Figure 3 shows the performance of our system on the test set. Each panel shows the rate at which interventions were recommended for a group as a function of time; the horizontal dashed green line shows the Caesarean delivery rate for that group. Figure 3.A shows that the recommendation rate for the normal group increased as delivery approached, but never exceeded the Caesarean delivery rate. Conversely, Figure 3.B and Figure 3.C show that the intervention recommendation rate for the acidosis and HIE groups surpassed the Caesarean rate at 2 h 40 min and 1 h 40 min respectively.

Table 1 displays the information in Figure 3 at the 20-min epoch ending 3 h before delivery, and the one ending 40 min before delivery. When comparing the Caesarean delivery rate to our results at the epoch ending 40 min before delivery, our system improved the detection of fetuses at risk of fetal acidosis in 10.7% (37.6% vs 48.3%, p<0.001) and those at risk of HIE by 6.9% (54.9% vs 61.8%, p<0.001). There was no increase in the intervention rate in the healthy group (38.9% vs 38.8%, p=0.26). Furthermore, 3/4 of these detections in the HIE group were made 3 h before delivery (47.4%/61.8%).

## 5. Discussion

The main finding in this work was that our prediction system recommended more and earlier Caesarean deliveries among infants who went on to develop HIE or acidosis when compared to the Caesarean rates in these groups. Furthermore, ¾ of the recommended interventions for the HIE group occurred 3 h or earlier before delivery as shown in Table 1. It is clinically reasonable to expect that an emergency Caesarean section performed this early prior to the actual delivery would improve outcome for some of these fetuses.

HIE, and its precursor fetal acidosis, are caused by sustained hypoxia. As delivery approaches, uterine contractions become more intense and frequent. FHR events such as decelerations are driven by the changes in blood pH and levels of oxygen. Figure 1 shows that the probability of categorizing the epochs as pathological increased in the three groups as delivery approached. Thus, it is of paramount importance to detect the fetal progression towards HIE as early as possible, such that preventive interventions could interrupt this process.

Aggregating consecutive predictions allowed our system to decide based on multiple predictions, which should reduce the effects of noise in single epochs. The best performance was obtained when requiring 5 consecutive alerts. Thus, our system requires a minimum of 1 h 40 min of monitoring before deciding. Although this might seem an excessively long delay, most of the individuals in our database had signals recorded for 12 h or longer, and the classifier was able to identify up to 61.8% of the HIE cases by the end of our testing protocol.

In a previous work, we developed a similar system that used 22 features of fetal heart rate variability (FHRV) for classification [7]. We detected 54.6% of acidosis and 60.2% of HIE individuals 1 h before delivery. Thus, the present work has the advantage of obtaining a similar detection of HIE while using fewer features. Furthermore, the present system takes advantage of machine learning to improve the interpretation of well-known features that clinicians already regard as physiologically relevant.

Finally, we note that it is difficult to compare our results to other studies in the literature. HIE is rare and consequently much previous work has used acidosis as a surrogate measure. Petrozziello et al. reported a TPR of 58% for a 20% FPR when training and testing on the last 60 minutes of CTG [8]. Their pathological class included those with severe compromise or not and pH < 7.05. Their healthy class included those with no compromise and pH ≥ 7.15. They removed from analysis those with pH values in between 7.05 to 7.15. Compared to them, we obtained similar TPR but with a higher FPR. However, our healthy group included any individual with pH > 7.0 if there was no evidence of encephalopathy or intrapartum complications. It is reasonable to expect that the FPR increases when including lower pH values in the healthy group. Thus, we cannot compare their FPR directly with our study. Furthermore, their predictions were made at the end of the CTG recordings [8]. Although the signals are more discriminatory at birth, it is also impossible to carry preventive interventions at that moment. Our system detected 47.4% of HIE cases at the epoch ending 3 h before delivery. These results are very promising for the goal of performing timely interventions that could lead to a reduction in the severe consequences of HIE.

## 6. Conclusion

This retrospective study demonstrated that the early detection of HIE from intrapartum FHR and UP signals can be improved without increasing unnecessary interventions. Furthermore, our system uses only a few features, all of them understandable to clinicians, which links our results to current clinical practice and knowledge. In future studies, we will focus on integrating these features to maternal and pregnancy clinical antecedents, which may further inform the classifier about the risk of the fetus developing intrapartum HIE.

## Figures and Tables

**Table 1.** Observed and recommended Caesarean delivery rates per group for the epochs starting 3 h 20 min and at 1 h before delivery. The values are the median and its 95% confidence interval.

Group	Caesarean Rate	Our System	
		*3 h 20 min*	*1 h*
Healthy	38.9% (38.7–39.1)	26.9% (26.8–27.1)	38.8% (38.7–39.0)
Acidosis	37.6% (37.0–38.2)	34.2% (33.7–34.7)	48.3% (47.8–48.9)
HIE	54.9% (53.3 – 56.4)	47.4% (46.0–48.9)	61.8% (60.4–63.3)
